# Prognostic Value of Interocular Flicker ERG Asymmetry in Acute Postoperative Endophthalmitis After Intraocular Surgery: A Pilot Study

**DOI:** 10.1155/joph/2982435

**Published:** 2026-06-30

**Authors:** Ying Zheng, Simon Dulz, Vasyl Druchkiv, Martin Stephan Spitzer, Christos Skevas

**Affiliations:** ^1^ Department of Ophthalmology, University Medical Center Hamburg-Eppendorf, Hamburg, Germany, uke.de; ^2^ Department of Ophthalmology, Shanghai General Hospital Affiliated to Shanghai Jiaotong University, Shanghai, China; ^3^ Department of Clínica Baviera, 3@R&D, Clínica Baviera, Valencia, Spain

**Keywords:** electroretinography, endophthalmitis, flicker ERG, inflammation, intraocular infection, prognosis

## Abstract

**Background/Objectives:**

Postoperative endophthalmitis remains a vision‐threatening complication despite advances in treatment. Current prognostic markers (Gram stain and initial visual acuity) have limited predictive values for real‐time retinal neuronal function assessment and visual recovery. The purpose of this pilot study was to explore whether photopic electroretinograms might be used to forecast the endophthalmitis prognosis following intravitreal injection or phacoemulsification intraocular lens implantation.

**Subjects/Methods:**

This prospective observational study included 11 consecutive patients with acute postoperative endophthalmitis following phacoemulsification with IOL implantation (3 eyes) or intravitreal injections (8 eyes). ERGs were recorded using a portable RETeval™ system under nonmydriatic conditions. R Core Team (2019) was used to assess the preoperative electroretinogram outcomes, duration until the onset of endophthalmitis, procedure types of original surgeries, and pre‐ and postoperative best‐corrected visual acuity.

**Results:**

Mean patient age was 79.23 years (range 62–88). A significant correlation was found between interocular photopic flicker latency difference and BCVA improvement (*p* = 0.04, < 0.05). Patients with ≤ 8 ms latency difference showed significantly better visual recovery (≥ 2‐line gain). No significant correlations were found with diabetes status, original surgical procedure types, or time to symptom onset.

**Conclusion:**

In this hypothesis‐generating pilot study of acute postoperative endophthalmitis, a smaller interocular flicker ERG latency difference at presentation is associated with better visual recovery. These preliminary findings suggest that this rapid, noninvasive functional test may have potential as a prognostic biomarker and warrants further validation in larger cohorts, complementing existing clinical and microbiological parameters in the management of this sight‐threatening infection.

## 1. Introduction

Postoperative endophthalmitis represents one of the most devastating complications of intraocular surgery, with potential for severe visual impairment despite prompt intervention [1]. Incidence rates vary according to surgical procedure, ranging from 0.033% to 0.082% after intravitreal injections to 0.06%–0.2% following cataract surgery [2, 3]. Given the rapid progression of retinal damage in endophthalmitis, early detection and appropriate management are critically important for visual preservation.

Current prognostic indicators primarily rely on microbiological factors and presenting visual acuity [4, 5]. While these parameters provide some predictive value, they offer limited information about real‐time retinal function and neuronal integrity. Animal studies have demonstrated that the b‐wave amplitudes of the scotopic electroretinogram (ERG) in *Enterococcus faecalis–* or *Pseudomonas aeruginosa*–induced endophthalmitis decreased within 2 or 3 days after inoculation [6–8]. However, translational clinical data regarding electroretinographic predictors in human endophthalmitis remain limited.

The aim of our study was to investigate whether photopic ERGs, measured using a portable nonmydriatic device, was able to predict the prognosis of the endophthalmitis after intraocular lens implantation or intravitreal injections.

## 2. Subjects and Methods

This prospective observational study was conducted at the Department of Ophthalmology, University Medical Center Hamburg‐Eppendorf, from January 2020 to December 2022. The study adhered to the tenets of the Declaration of Helsinki and received approval from the Hamburg Ethics Committee (2021‐100631‐BO‐ff). Written informed consent was obtained from all participants.

Consecutive patients presenting with acute postoperative endophthalmitis following either phacoemulsification with intraocular lens implantation or intravitreal injections were screened for eligibility. Endophthalmitis diagnosis required the presence of characteristic clinical signs and symptoms (ocular pain, decreased vision, eyelid edema, conjunctival congestion, chemosis, anterior chamber inflammation, hypopyon, vitritis, or diminished red reflex) within 1 week of intraocular surgery. Patients with other reasons for endophthalmitis (endogenous source, posttrauma, postfiltrating surgery, and post‐PPV) and patients treated with vitreous tap/biopsy were excluded. The criteria employed to diagnose endophthalmitis were fundoscopy, ultrasound with vitreous body infiltration, pain, hypopyon, anterior chamber inflammation, and medical history. Recorded parameters were patient‐related data, pre‐existing general health conditions, endophthalmitis‐related data, duration until the onset of symptoms or signs, best‐corrected visual acuity (BCVA) measurement using Snellen charts (converted to logMAR for analysis), ERGs, and treatment.

Electroretinography was performed using the RETeval™ system (LKC Technologies, Gaithersburg, MD) under nonmydriatic conditions (pupil size 2–4 mm). Skin electrodes were positioned following manufacturer guidelines. Flicker ERGs were elicited by white light at 28.3 Hz frequency and 8 Td‐s intensity, corresponding to the device’s default setting for nondilated eyes. No background adaptation light was used in this study. The contralateral eye was covered during testing. Amplitude and implicit time values of the fundamental component were automatically analyzed and exported using the integrated RETeval™ software.

All patients underwent standardized 23‐gauge pars plana vitrectomy with intravitreal antibiotic injection (vancomycin 1 mg/0.1 mL and ceftazidime 2.225 mg/0.1 mL). No patients received intravitreal voriconazole during initial surgery. Vitrectomy did not include peripheral vitreous base shaving, and posterior vitreous detachment was not induced as all cases showed pre‐existing PVD according to surgical reports. After completion of the vitrectomy, a thorough but not too forceful examination of the peripheral retina was performed in order to locate any retinal breaks [9].

### 2.1. Ethical Approval

The study adhered to the tenets of the Declaration of Helsinki. The study has been reviewed and approved by the ethics committee of Hamburg (2021‐100631‐BO‐ff).

### 2.2. Statistical Analysis

All statistical calculations were performed using R Core Team (2019). The correlation between any two continuous variables was measured and tested using Spearman’s correlation coefficient. The differences in visual acuity and latency between the factor levels were tested using the Mann–Whitney test, as the assumptions of the parametric *t* test were not satisfied in any of the comparisons. Statistical significance was set at *p* < 0.05.

## 3. Results

This prospective observational study included 11 eyes of 11 patients with postoperative endophthalmitis following intraocular lens implantations or intravitreal injections. The mean age was 79.23 years (range 62–88 years). Five patients (45%) were male, and six patients (55%) were female.

**FIGURE 1:**
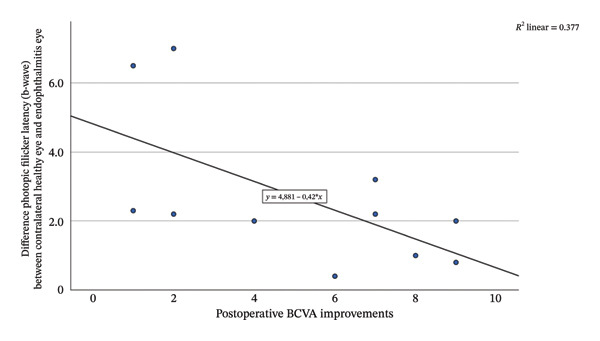
Correlation between the difference in photopic flicker latency (b‐wave) between the contralateral healthy eye and endophthalmitis eye and the postoperative BCVA improvement.

Baseline BCVA was 1.8 ± 0.6 logMAR (approximately 20/1260 Snellen equivalent), improving to 0.9 ± 0.7 logMAR (approximately 20/160 Snellen) at final follow‐up. The interocular photopic flicker ERG latency difference (b‐wave) showed a significant correlation with BCVA improvement (*ρ* = −0.62, *p* = 0.04; Figure 1). Regression analysis revealed a substantial linear relationship (*y* = 4.81 − 0.42*x*, *R* ^2^ = 0.377), indicating that each 1‐ms reduction in latency difference corresponded to 0.42 lines of BCVA improvement. The smaller the difference in photopic flicker latency (b‐wave) between the contralateral healthy eye and endophthalmitis eye, the greater the postoperative BCVA improvement. Receiver operating characteristic (ROC) analysis identified a latency difference threshold of ≤ 8 ms as predictive of ≥ 2‐line BCVA improvement, with an area under the curve (AUC) of 0.81 (95% confidence interval: 0.52‐0.98), sensitivity of 83%, and specificity of 75%. The wide confidence interval reflects the limited sample size.

**FIGURE 2:**
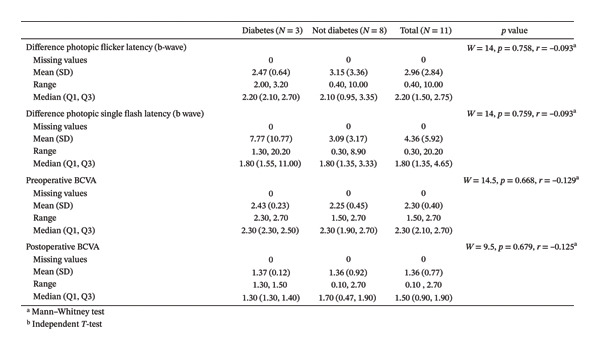
Variables by diabetes.

Out of these 11 patients, three (27.27%) were diagnosed of diabetes. There were no significant correlations between the diabetes group and none diabetes group in photopic flicker latency (b‐wave), photopic single flash latency (b‐wave), and pre‐/postoperative BCVA (*p* = 0.758, 0.759, 0.668, 0.679, respectively, and *r* = 0.093, 0.093, 0.129, and 0.125, respectively) (Figure 2). The median interocular flicker latency difference was 2.20 ms (Q1–Q3: 1.50–2.75) in the overall cohort, with similar values between diabetic (2.20 ms; Q1–Q3: 2.10–2.70) and nondiabetic patients (2.10 ms; Q1–Q3: 0.95–3.35).

**FIGURE 3:**
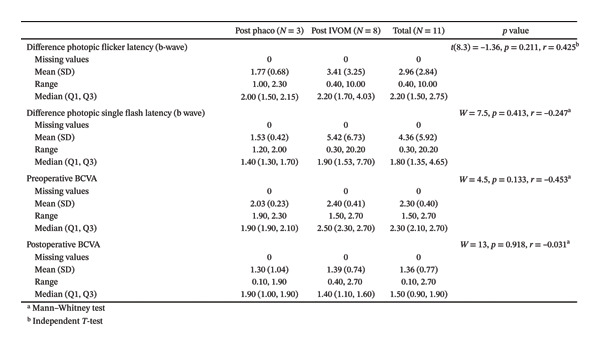
Variables by procedure types of original surgeries.

The original surgical procedures which resulted in the endophthalmitis were phacoemulsification and intraocular lens implantation in three eyes (27.27%) and intravitreal injections in eight eyes (72.73%). There were also no significant correlations between the phaco group and the intravitreal injection group in photopic flicker latency (b‐wave), photopic single flash latency (b‐wave), and pre‐/postoperative BCVA (*p* = 0.211, 0.413, 0.133, and 0.918, respectively, and *r* = 0.425, 0.247, 0.453, and 0.031, respectively) (Figure 3).

**FIGURE 4:**
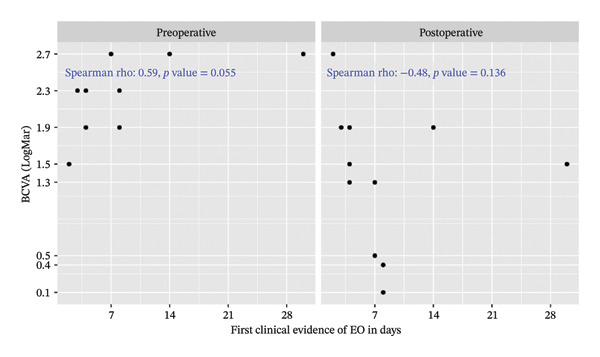
Correlation between the duration of the onset of endophthalmitis and the pre‐/postoperative BCVA.

**FIGURE 5:**
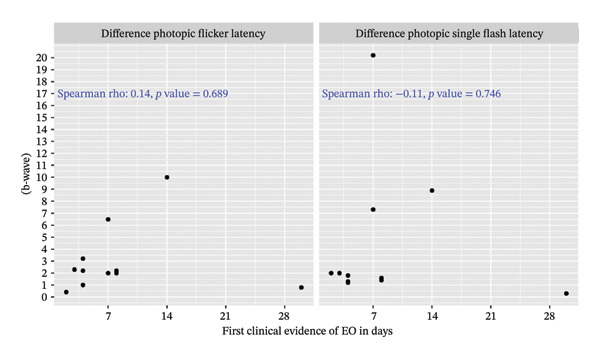
Correlation between the duration of the onset of endophthalmitis and the photopic flicker latency, photopic single flash latency.

First clinical evidence of endophthalmitis was reported in median 7 days after the original surgeries (Q25: 4 days, Q75: 8 days). There were no significant correlations between the duration of the onset of endophthalmitis and the pre‐/postoperative BCVA (*p* = 0.59 and 0.48, respectively, and *r* = 0.055 and 0.136, respectively) (Figure 4). Moreover, there were no significant correlations between the duration of the onset of endophthalmitis and the photopic flicker latency, photopic single flash latency as well (*p* = 0.689 and 0.746, respectively, and *r* = 0.14 and −0.11, respectively) (Figure 5).

Vitreous cultures were obtained from all 11 patients (100%). All 11 cultures (100%) were positive for *Staphylococcus aureus*. No other bacterial or fungal species were isolated.

## 4. Discussion

Exogenous endophthalmitis following intraocular surgeries can lead to vision‐threatening consequences. Of the different types of postoperative endophthalmitis, acute endophthalmitis accounts for most cases (88%). Acute endophthalmitis usually appears within one to 2 weeks after the primary surgical interventions (the median onset of postoperative endophthalmitis after cataract surgeries: 9 days; postintravitreal injection: 24 h) [2, 3]. The early diagnosis and proper treatment are clinically important.

Numerous studies attempted to predict the prognosis of postoperative endophthalmitis. It was reported that a positive Gram stain or infection with species other than Gram‐positive, coagulase‐negative micrococci were significantly associated with poorer visual outcome, and moreover, visual acuity at initial presentation appeared to be more useful in predicting visual outcome and judging the value of immediate vitrectomy in acute bacterial endophthalmitis after cataract surgery [5]. Lee et al. also showed that advanced age and initial visual acuity were risk factors for a poor visual prognosis in cases of endophthalmitis caused by various etiologies [4]. Despite these important results, these studies could not yet provide more information on the impairment of retinal function.

The ERG is an electrical response of the retina to photic stimulation. The a‐wave which originates at the receptor level of rods and cones reflects the general physiologic health of the photoreceptors in the outer retina, while the b‐wave which originates from the midretina reflects the health of the inner layers of the retina, including the ON bipolar cells with contributions from the other midretinal cells, such as horizontal and amacrine cells [10, 11]. In some ocular fundus diseases, significant ERG changes could be observed. For example, ERG changes associated with central retinal vein occlusion are attenuation of b‐wave amplitude and delay of 30‐Hz flicker implicit time to beyond 35 ms [12]. Our pilot study suggests that interocular asymmetry in photopic flicker ERG latency may serve as a potential predictor of visual recovery in acute postoperative endophthalmitis. Smaller latency differences (≤ 8 ms) were associated with better visual outcomes, suggesting preserved retinal neuronal function despite infection, which might, after validation, assist clinicians in identifying patients who may benefit from early vitrectomy versus those who might be managed more conservatively. The RETeval™ system used in our study offers several practical advantages for endophthalmitis assessment. Its portability enables bedside testing in emergency settings (< 5 min), while nonmydriatic operation facilitates rapid evaluation without pharmacological delay. The automated analysis provides objective results within minutes, contrasting with conventional ERG systems requiring technical expertise and prolonged testing sessions. This finding represents a novel application of portable ERG technology for bedside prognostication in ocular infections.

Kim et al. reported that rabbit eyes infected with *P. aeruginosa* showed a significant decrease in b‐wave amplitudes after 24 h, resulting in flat ERG b‐waves [8]. Our results align with these previous animal studies in experimental endophthalmitis but extend these findings to human patients using contemporary recording technology. Horio et al. demonstrated that a rapid decrease in b‐wave amplitudes was observed in the eyes infected of highly virulent organisms and that a b/a amplitude ratio less than 1.0 and early onset of endophthalmitis within 1 week might indicate a poor prognosis in endophthalmitis after intraocular lens implantation [11]. However, in our study, the difference in photopic flicker latency (b‐wave) between the contralateral healthy eye and endophthalmitis eye was significantly associated with the visual prognosis, instead of the decreased amplitudes or the b/a amplitude ratio. Since lid muscle artifact often attenuates the amplitude of 30‐Hz flicker ERGs due to the irritating bright flashes, a low‐amplitude 30‐Hz flicker response is not an accurate reflection of cone physiology [10]. Moreover, our findings align with the contemporary understanding of retinal neurophysiology that emphasizes the robustness of latency measurements. The 28.3‐Hz flicker stimulus specifically targets the cone‐pathway function, which appears particularly sensitive to the inflammatory environment of endophthalmitis. This technical superiority explains why we detected significant prognostic values in latency measurements where previous studies using amplitude parameters yielded inconsistent results.

Patients with diabetes usually have an impaired immune response and tend to get surgical infections, since hyperglycemia activates the polyol pathway of glucose metabolism and generates cellular stress and apoptosis of vascular pericytes and endothelial cells [12, 13]. EVS study reported that 55% of patients without diabetes achieved visual acuity of at least 6/12 compared with 39% of diabetic patients [14]. Zakereia et al. reported that there was a significant increase in b‐wave latency in the diabetic retinopathy group compared with the diabetics‐without‐complication group and the control group, while there was no significant difference in b‐wave latency between the diabetics‐without‐complication group and the control group [15]. This was consistent with our study results that there were no significant correlations between the diabetes group and none diabetes group in photopic flicker latency (b‐wave), photopic single flash latency (b‐wave), and pre‐/postoperative BCVA. Theses controversial results might result from the difference in glycemic control, duration of diabetes, and the presenting vision acuity.

It was reported that the incidence rates of postoperative endophthalmitis range differently due to different primary surgical interventions (e.g., postcataract surgery: 0.06%–0.2%; postintravitreal injection: 0.033%–0.082%) [2, 3]. Horio et al. demonstrated that the a b/a amplitude ratio less than 1.0 might indicate a poor prognosis in endophthalmitis after intraocular lens implantation [11]. However, in our study, we found that there were no significant correlations between the phaco group and the intravitreal injection group in photopic flicker latency (b‐wave), photopic single flash latency (b‐wave), and pre‐/postoperative BCVA. This might be due to the smaller number of patients in the phaco group. Although a larger number of cases need to be included for further investigations of the correlation between ERG results and different primary surgical interventions, the prognostic value of the interocular flicker latency difference was maintained across different surgical procedures, as well as across both diabetic and nondiabetic subgroups. This consistency suggests that the pathophysiological processes affecting retinal neural conduction in endophthalmitis might transcend these variables.

Acute endophthalmitis usually appears within one to 2 weeks after the primary surgical interventions (the median onset of postoperative endophthalmitis after cataract surgeries: 9 days; postintravitreal injection: 24 h) [16, 17]. Horio et al. demonstrated that early onset of endophthalmitis within 1 week might indicate a poor prognosis in endophthalmitis after intraocular lens implantation [11]. In our study, the first clinical evidence of endophthalmitis was reported in median 7 days after the original surgeries (Q25: 4 days, Q75: 8 days), and statistical results showed that there were no significant correlations between the duration of the onset of endophthalmitis and the pre‐/postoperative BCVA. These results might be due to the different primary surgical interventions (The original surgical procedures which resulted in the endophthalmitis were phacoemulsification and intraocular lens implantation in three eyes and intravitreal injections in eight eyes). On the other hand, these results might challenge conventional assumptions about the temporal progression of endophthalmitis and emphasize the importance of assessing retinal function directly rather than relying solely on temporal factors.

The endophthalmitis vitrectomy study demonstrated that causative organisms strongly influence visual outcomes, with more virulent pathogens (e.g., Streptococcus, Enterococcus, and Gram‐negative bacilli) associated with a poorer prognosis [5]. In our study, all culture‐positive cases (11/11, 100%) were caused by *Staphylococcus aureus*—a pathogen with well‐characterized intermediate virulence. The uniformity of the causative organism across all patients eliminates pathogen type as a potential confounder when interpreting the association between interocular ERG latency difference and visual recovery. This homogeneity substantially strengthens the internal validity of our finding that smaller latency differences predict better visual outcomes. Nevertheless, the small sample size precludes definitive conclusions, and future studies should include a broader spectrum of pathogens to assess whether the prognostic value of the ERG latency difference varies by organism virulence.

A major limitation is the absence of serial ERG recordings following treatment initiation. In this study, ERG was performed only at presentation (baseline). Serial measurements, particularly after vitrectomy and antibiotic administration, could provide valuable insights into the recovery trajectory of retinal function and whether ERG changes track with clinical improvement. Such longitudinal data would strengthen the causal inference between retinal neuronal dysfunction and visual outcomes. Future prospective studies could incorporate serial ERG assessments (e.g., at Day 3, Week 1, and Month 1 posttreatment) to evaluate the dynamic relationship between electrophysiological recovery and final visual acuity. Other limitations are the small sample, lack of a defined treatment protocol, treatment by multiple vitreoretinal surgeons, and exclusion of cases due to the complexity of the disease. The heterogeneous surgical procedures, while reflecting real‐world clinical practice, may introduce confounding factors. Therefore, larger number of cases and multiple centers need to be included for further studies in order to provide more information on this complex issue, especially the information on whether ERG parameters correlate with specific microbiological isolates or inflammatory markers.

In conclusion, this pilot study provides preliminary evidence that the interocular flicker ERG latency difference may be associated with visual recovery and could be explored as a prognostic marker in acute postoperative endophthalmitis. Portable ERG systems enable rapid bedside assessment that could inform treatment decisions and improve visual outcomes. Validation in larger cohorts may establish this technology as a standard tool for endophthalmitis management.

## Author Contributions

Christos Skevas designed the research, Ying Zheng and Simon Dulz collected the data, Vasyl Druchkiv did the statistics, Martin Stephan Spitzer and Christos Skevas revised the manuscript, and Ying Zheng and Simon Dulz wrote the manuscript.

## Funding

Open access funding was enabled and organized by Projekt DEAL.

## Disclosure

Ying Zheng and Simon Dulz are both co‐first authors. All authors read and approved the final manuscript.

## Ethics Statement

All procedures performed in the study involving human participants were in accordance with the ethical standards of the institutional and/or national research committee and with the 1964 Helsinki Declaration and its later amendments or comparable ethical standards. The study has been reviewed and approved by the ethics committee of Hamburg (2021‐100631‐BO‐ff). Written informed consent was obtained from all participants.

## Conflicts of Interest

The authors declare no conflicts of interest.

## Data Availability

The data that support the findings of the study are available from the corresponding author upon reasonable request.
